# A causal role for the right angular gyrus in self-location mediated perspective taking

**DOI:** 10.1038/s41598-020-76235-7

**Published:** 2020-11-05

**Authors:** D. M. L. de Boer, P. J. Johnston, G. Kerr, M. Meinzer, A. Cleeremans

**Affiliations:** 1 School of Psychology and Counselling, Faculty of Health, Queensland University of Technology (QUT), Kelvin Grove, QLD 4059 Australia; 2 School of Exercise and Nutrition Sciences, Faculty of Health, Queensland University of Technology (QUT), Kelvin Grove, QLD 4059 Australia; 3 Institute of Health and Biomedical Innovation (IHBI), Queensland University of Technology (QUT), Brisbane, Australia; 4 Centre for Clinical Research (UQCCR), The University of Queensland, Brisbane, Australia; 5 Department of Neurology, University Medicine Greifswald, Greifswald, Germany; 6 Consciousness, Cognition, and Computation Group (CO3), Centre for Research in Cognition and Neurosciences (CRCN), ULB Neuroscience Institute (UNI), Université Libre de Bruxelles (ULB), Avenue F.D. Roosevelt 50, CP191, 1050 Brussels, Belgium

**Keywords:** Neuroscience, Psychology

## Abstract

Recent theories suggest that self-consciousness, in its most elementary form, is functionally disconnected from the phenomenal body. Patients with psychosis frequently misattribute their thoughts and actions to external sources; and in certain out-of-body experiences, lucid states, and dreams body-ownership is absent but self-identification is preserved. To explain these unusual experiences, we hypothesized that self-identification depends on inferring self-location at the right angular gyrus (i.e., perspective-taking). This process relates to the discrimination of self-produced signals (endogenous attention) from environmental stimulation (exogenous attention). Therefore, when this mechanism fails, this causes altered sensations and perceptions. We combined a Full-body Illusion paradigm with brain stimulation (HD-tDCS) and found a clear causal association between right angular gyrus activation and alterations in self-location (perspective-taking). Anodal versus sham HD-tDCS resulted in: a more profound out-of-body shift (with reduced sense of agency); and a weakened ability to discriminate self from other perspectives. We conclude that self-identification is mediated in the brain by inferring self-location (i.e., perspective-taking). Self-identification can be decoupled from the bodily self, explaining phenomena associated with disembodiment. These findings present novel insights into the relationship between mind and body, and may offer important future directions for treating psychosis symptoms and rehabilitation programs to aid in the recovery from a nervous system injury. The brain’s ability to locate itself might be the key mechanism for self-identification and distinguishing self from other signals (i.e., perspective-taking).

## Introduction

**Figure 1:**
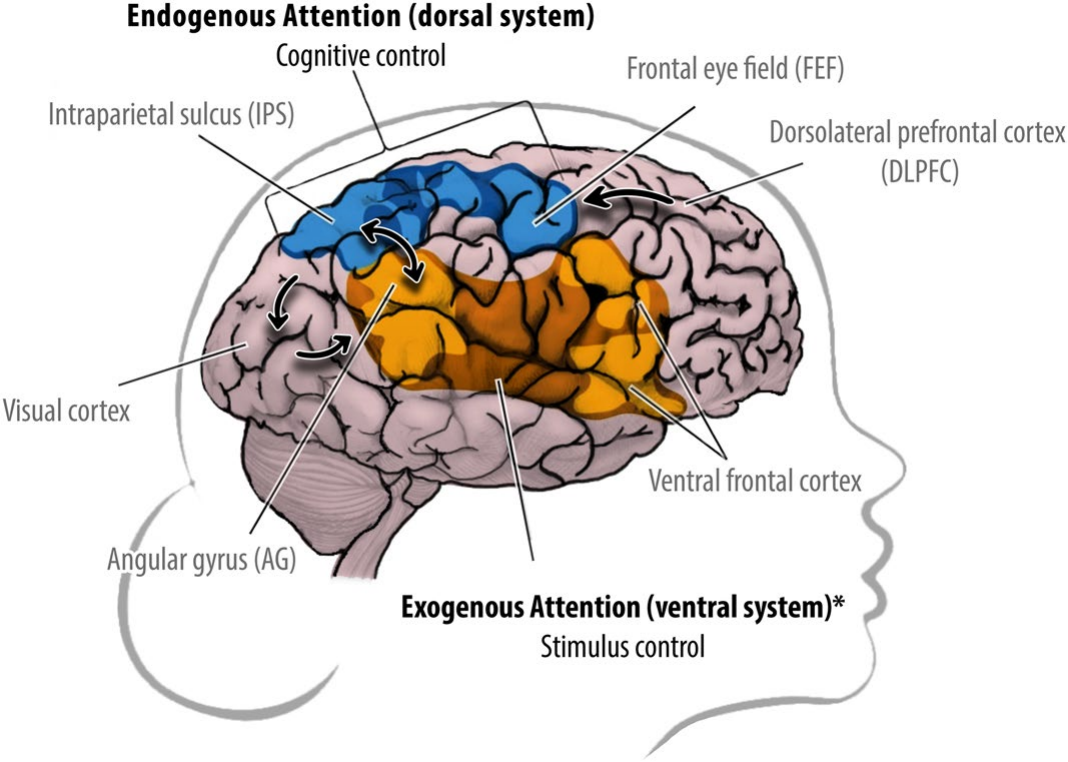
**Endogenous (dorsal) versus exogenous (ventral) attention systems.** Dorsal (blue) and ventral (yellow) frontoparietal attention systems (as outlined by^14^). Humans can perceive the world from different perspectives: an endogenous (Self) versus exogenous (Other) focus of attention. Self-produced signals are predicted in the brain (e.g., efference copies), whereas external signals are not. These predictions allow the brain to early discriminate self-produced signals from external stimulation and keep track of its own spatiotemporal location (perspective). **Endogenous attention**: the dorsal system is goal-directed and processes self-produced signals. ‘Cognitive control’ enables the top-down voluntary selection of stimuli and responses, resp.: dorsolateral prefrontal cortex (dlPFC), frontal eye field (FEF), intraparietal sulcus (IPS) and visual cortex. **Exogenous attention** * lateralized to the right hemisphere: the ventral system is stimulus-driven and processes external signals. ‘Stimulus control’ enables the automatic orientation and bottom-up detection of behaviorally relevant stimuli, resp.: visual cortex, angular gyrus (AG; part of the temporoparietal junction) and the ventral frontal cortex. These attention systems keep external and self-produced signal processing separated and interconnect at the right AG (double arrow). **Bidirectional mechanism**: at any given time, the right AG may orient attention outwards to salient or unexpected external events, acting as a ‘circuit breaker’ for the dorsal system; in return, when the dlPFC detects self-produced signals AG’s function is inhibited, silencing the ventral system. When this decoupling fails, self-produced signals are processed as if uncontrolled by oneself (e.g., delusion of control^4^); and (over)stimulation of the right AG (e.g., electrical stimulation, epileptic seizures) results in signals being processed as if they are located outside the body (i.e., out-of-body experience). Endogenous = ‘of internal origin;’ exogenous = ‘of external origin.’ . Figure redrawn from^13^.

Recent theories suggest that self-consciousness, in its most elementary form, is functionally disconnected from the sense of owning a body (see *Minimal Phenomenal Selfhood*^1,2^). In out-of-body experiences (OBEs) subjects experience themselves as being located outside of their physical bodies^3^; and in hallucinations and delusions people often misattribute their thoughts and actions to external sources (see *psychosis*^4^). Self-identification can even persist when no explicit body representation is present (e.g., bodiless dreams/OBEs), leading to the conclusion that the phenomenal body is not a minimal condition for being self-conscious(^1,2^; cf.^5^). We propose a bidirectional neural mechanism to help explain why, under certain conditions, humans can perceive themselves as being disconnected from their own bodies and body-parts (i.e., disembodied as opposed to embodied agents). Previous studies have shown that electrical stimulation of the right angular gyrus induces out-of-body experiences (^6,7^ and Penfield in^3^; for a meta-analysis see^8^); and reduces the recognition of self-produced signals and self-other discrimination(^9–11^; also see^12^). However, what connects these findings remains unclear. We hypothesized that the phenomena associated with disembodiment arise by unusual right angular gyrus activation when the brain fails to identify and discriminate self-produced signals from external stimulation (resp. *endogenous vs. exogenous attention*^13^). Abnormal sensory processing can lead to aberrant self-other reference frames (i.e., perspective-taking), and, in more extreme cases, unusual self-location, Fig. 1. Clues for this mechanism can be found in psychopathological cases of agency^15–18^.

The brain’s ability to recognize and discriminate self-produced signals from external stimulation is tied to the process of sensory gating. Sensory information is transformed in the brain through a complex system of gating steps that filter out irrelevant noise and excessive sensory information^19^. When lifting your arm, for instance, neural representations or ‘copies’ of motor commands are generated. Efference copies travel to the sensory cortex and attenuate feedback stemming from our own sensory systems (see *comparator models*^20^). Sensory attenuation^21^ thus informs the brain whenever it is stimulating itself, and accounts for why we cannot tickle ourselves^20,22^ and why we are unaware of our eye-saccades^23,24^. In psychosis these systems appear to be compromised^19^. Recent studies^15–18^ show that people who experience hallucinations and delusions are unable to monitor self-produced signals. These findings indicate that a defect in predicting one’s own body signals causes the brain to not recognize being in control of them (see *sense of agency*^25,26^), resulting in altered sensations and perceptions. But how might this function on a systemic level? Interestingly, recent imaging studies that looked at abnormal agency in psychosis(^18^; also see^27^) and movement disorders^28^ reported decreased structural and altered functional connectivity between the frontoparietal control network and the right angular gyrus. Furthermore, a recent meta-analysis of tDCS studies found a causal link between agency processing and action selection in the dorsolateral prefrontal cortex (dlPFC)^29^. Specifically, the decoupling between the dlPFC (endogenous attention) and the right angular gyrus (exogenous attention^13^) appears to be impaired in psychosis: when the brain selects a voluntary movement, the inhibitory control of the frontal cortex to the right angular gyrus is lost^18^. Altered sensory processing is thus what leads to unusual perceptions. These recently overlooked findings shed light on the origin of disembodiment experiences and neural basis of self-identification. The brain’s ability to locate itself might be the key mechanism for self-identification and distinguishing self from other signals (i.e., perspective-taking).

To examine this hypothesis, we combined a full-body illusion paradigm with brain stimulation using high-definition tDCS. In a typical full-body illusion people observe a virtual body a few feet in front of them through a head-mounted display (HMD). For a few minutes they see what they feel happening to them (e.g., back stroking) play out in front of them. This leads people to identify with the virtual body and judge their spatial location to be closer to the external body than their actual location^30,31^. However, because of safety and compatibility issues it has been difficult to combine HMDs with neuroscience tools and elucidate the neural mechanisms underlying such illusions^8,32^. To address this problem, we developed a stereoscopic 3D-projection of a full-body illusion: video-captured images were LIVE-streamed to a computer and in real-time merged and projected onto a large screen. This allowed us to systematically manipulate and compare the brain function of healthy volunteers that (1) underwent a full-body illusion, and (2) carried out a perspective-taking task. We expected that anodal (vs. sham) right angular gyrus stimulation would: (1) make individuals feel more localized towards the virtual body; while (2) reducing their ability to discriminate between perspectives. Our results suggest that self-location (perspective-taking) is the key mechanism of self-identification.

## Results

The experiment had two sessions that each included a (1) full-body illusion (FBI) paradigm; and a (2) self-other perspective-taking (PT) task (i.e., own-body Transformation (OBT) vs. control Lateralization (LAT) task^33^). The two tasks were randomized and counterbalanced over HD-tDCS conditions. This resulted in four experimental conditions: FBI-PT with active stimulation on Session 1 (FBI-PT 1) or Session 2 (FBI-PT 2); and PT-FBI with active stimulation on Session 1 (PT-FBI 1) or Session 2 (PT-FBI 2). Sham stimulation was presented on all other occasions. Thirty-six prescreened participants (see “Materials and methods”) were randomly assigned to one of the four conditions, resulting in nine participants in each group. One participant was excluded from the FBI-analysis due to unforeseen technical problems (group PT-FBI 1; female; FBI *N* = 35). Two other participants were excluded from the PT-analysis because of chance-level performance (group PT-FBI 1 and FBI-PT 2; females; OBT and LAT *N* = 34). Occasional outliers in reaction times (< 1% data points LAT: RT < 160 ms/> 1000 ms; OBT: RT < 200 ms/ > 2000 ms) and missing values (< 3% data points PT-task) were replaced by mean values. Statistical analyses were performed in SPSS v25.0 and are reported using a 0.05 significance level.

### Full-body illusion (FBI)

Statistics pooled over the sessions revealed that the average reported displacement towards the projected image was 69.1 cm in Session 1 and 72.4 cm in Session 2. Participants reported that the displacement occurred “*After a while*” (*N* = 22 Session 1; *N* = 19 Session 2) or after 3 min (*M* = 3.6, *SD* = 1.8). This was experienced “*Many short times*” (*N* = 15 Session 1; *N* = 17 Session 2) followed by “*Continuously*” (*N* = 5 Session 1; *N* = 8 Session 2) and “*Once shortly*” (*N* = 7 Session 1; *N* = 2 Session 2). Maximal displacement was reported on nine occasions (180–200 cm); while 1/6 of participants reported no displacement (17.1%). The exit-interview consisted of 15 items (incl. three control items), answered on 5-point Likert scales ranging from ‘*1* = *Strongly Disagree*’ to ‘*5* = *Strongly Agree.*’ The items measured: ‘*Displacement*,’ ‘*Self-Identification*,’ and ‘*Sense-of-Agency*’ (see “Supplementary Materials”). Most prominently, participants perceived the displacement as: (1) a “*loss of control*” (Item 8, Agency low; *N* = 24 Session 1, *N* = 25 Session 2); followed by (2) a sense of dissociation from the bodily self “*I felt a shift out of my body towards the virtual body*” (Item 2, Main Displacement; *N* = 19 Session 1, *N* = 23 Session 2); and (3) subsequent identification with the virtual self (Items 5, 6, 10, and 15; Self-Identification; *M*_*tot*_ = 15 Session 1, *M*_*tot*_ = 15.6 Session 2). A large subset also reported (4) a regain in control “*I felt as if I could stand up and walk away with the virtual body*,” i.e., adopting a first-person perspective in the illusion (Item 11, Agency high; *N* = 14 Session 1, *N* = 15 Session 2). “*Total Exit Interview Scores*” were calculated excluding the control items.

**Figure 2:**
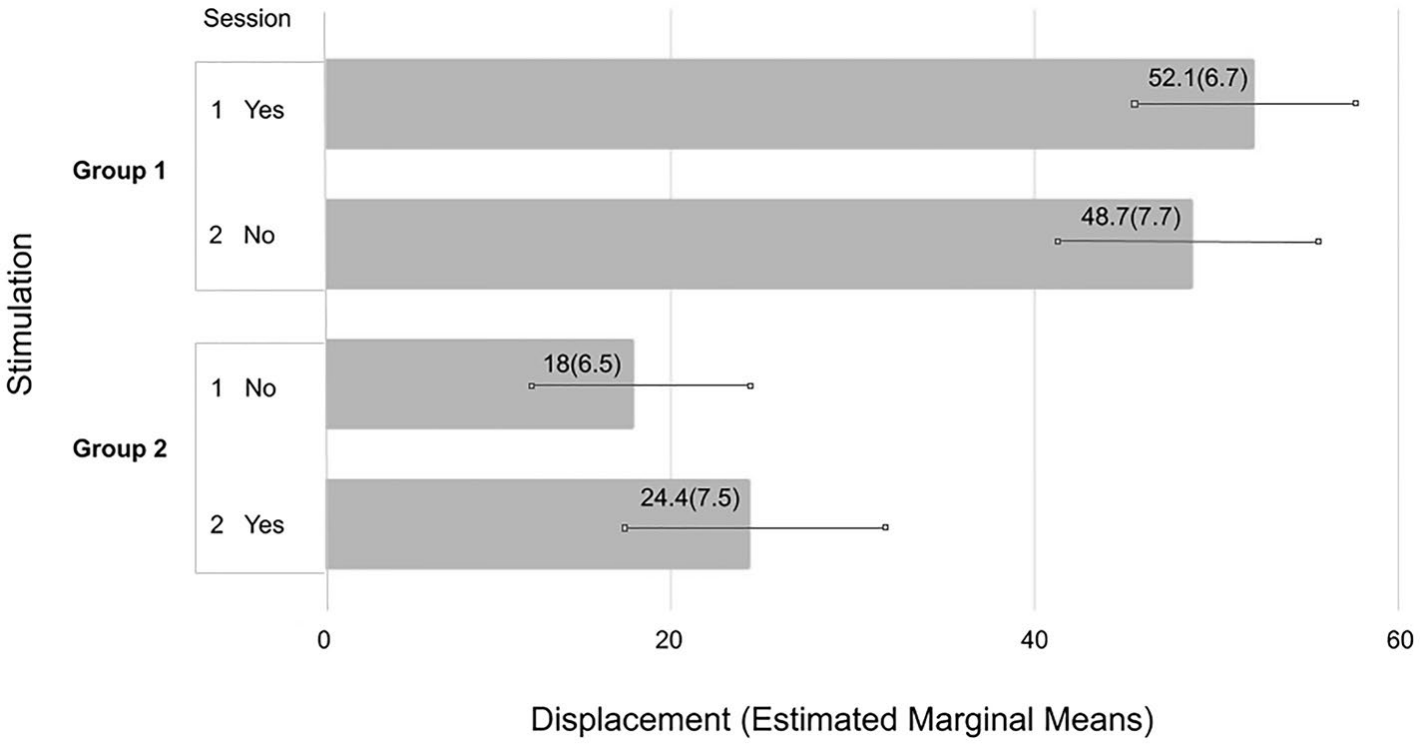
Behavioral measure: full-body illusion displacement * stimulation. Reported ‘Displacement’ by ‘Group 1’ active stimulation on Session 1 ‘Yes’ vs. Session 2 ‘No’ and ‘Group 2’ active stimulation on Session 1 ‘No’ vs. Session 2 ‘Yes.’ Estimated Marginal Mean (Standard Error) Bonferroni corrected; Group 1: FBI-PT 1 and PT-FBI 1 (*N* = 17); Group 2: FBI-PT 2 and PT-FBI 2 (*N* = 18).

**Figure 3:**
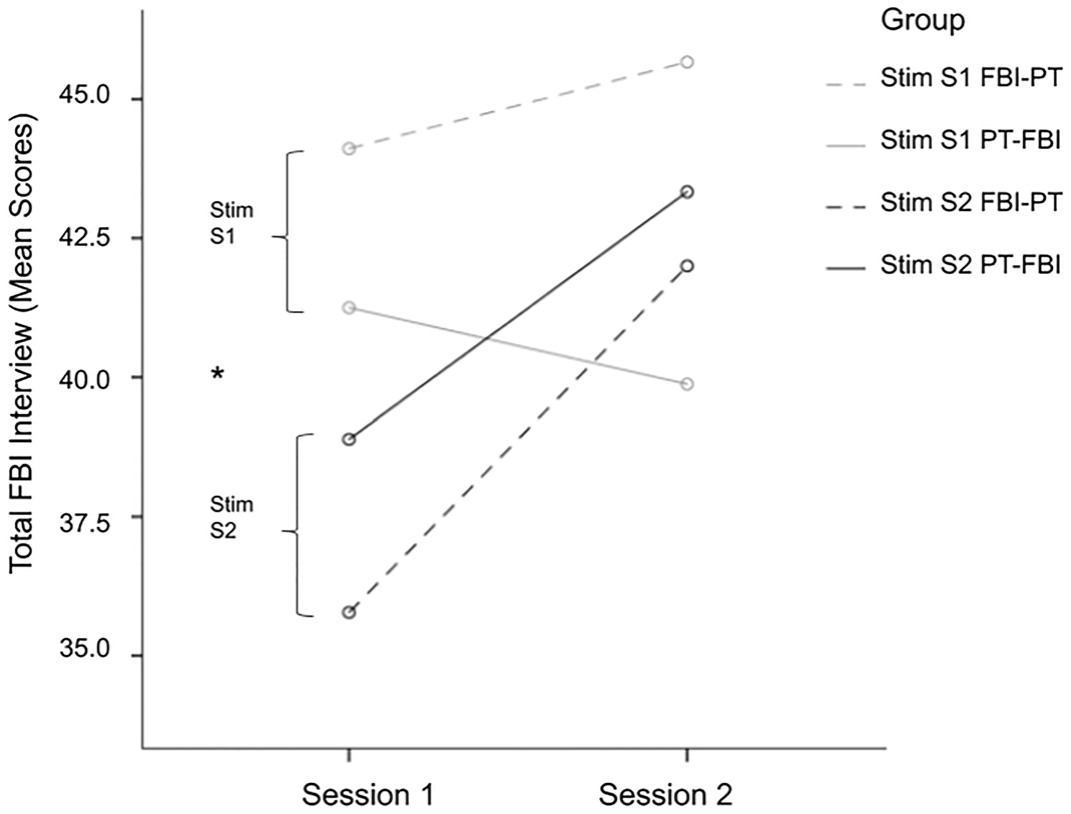
Psychometric measure: full-body illusion exit interview * stimulation. Mean Total Exit Interview scores per Session plotted by Group: solid and dashed grey lines received active stimulation on Session 1 (FBI-PT 1 N = 9 and PT-FBI 1 N = 9); solid and dashed black lines received active stimulation on Session 2 (FBI-PT 2 N = 8 and PT-FBI 2 N = 9). *Stim* stimulation, *S1* session 1, *S2* session 2, *FBI* full-body illusion, *PT* perspective taking; *p*-value < 0.05 = * (Groups: Stim S1 *N* = 18 vs. Stim S2 *N* = 17).

As expected, a first mixed ANOVA found a significant main effect for displacement in pre- and posttest scores over sessions, *F*(1,31) = 62.8, *p* < 0.001, ηp^2^ = 0.67 (behavioral measure). A second mixed ANOVA performed over “*Total Exit Interview Score*” also indicated a significant main displacement effect over sessions, *F*(1,31) = 5.2, *p* = 0.03, ηp^2^ = 0.14 (psychometric measure). These results confirmed that the new 3D FBI-paradigm had been successful. Furthermore, a significant effect of HD-tDCS to the right angular gyrus was found. The previous mixed ANOVA measured a significant interaction in displacement scores between experimental groups, *F*(3,31) = 4.4, *p* = 0.01, ηp^2^ = 0.30. There was no specific hypothesis based on task order (FBI-PT vs. PT-FBI), nor did Levene’s test for homogeneity indicate unequal group variances, *F* < 1. Therefore, in subsequent analyses the results were taken together comparing two groups differing in stimulation order. These analyses revealed a significant interaction between displacement scores and stimulation, *F*(1,33) = 10.5, *p* = 0.003, ηp^2^ = 0.24 (Fig. 2); as also between “*Total Exit Interview Scores*” and stimulation, *F*(1,33) = 4.8, *p* = 0.035, ηp^2^ = 0.13 (Fig. 3). Group 1: HD-tDCS S1 ‘*Yes*’ *M* = 42.8(6.4) vs. S2 ‘*No*’ *M* = 42.9(7.1); Group 2: HD-tDCS S1 ‘*No*’ *M* = 37.3(8.3) vs. S2 ‘*Yes*’ *M* = 42.7(8.4), Bonferroni corrected. Note: to provide more details Fig. 3 includes the original four groups. In short, both the behavioral and psychometric measure confirmed that more displacement was reported when the right angular gyrus received active versus sham stimulation. Moreover, a strong correlation between the amount of displacement and interview scores was found, *r*(33) = 0.57, *p* < 0.01 (Session 1) and *r*(33) = 0.51, *p* < 0.01 (Session 2), one-tailed Bonferroni corrected.

### Perspective taking (OBT and LAT tasks)

**Figure 4:**
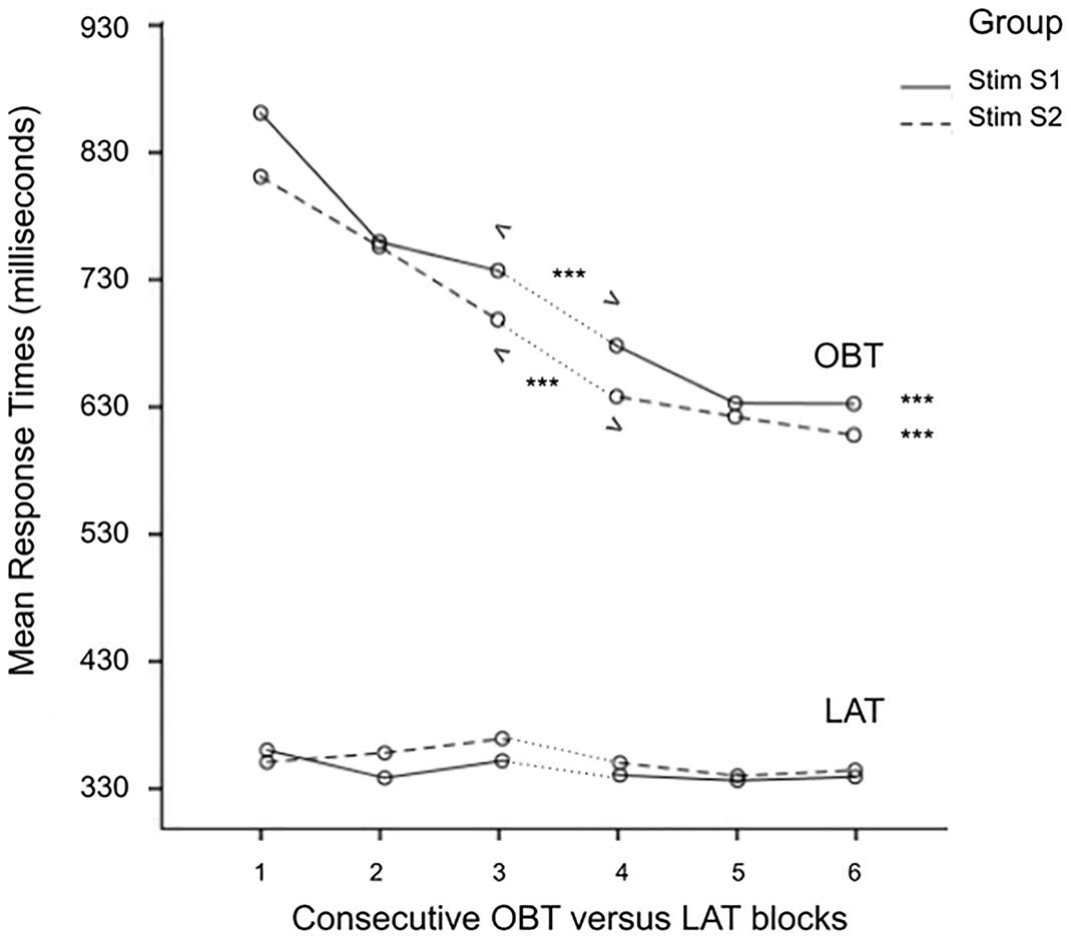
**Improvement in mean response times (milliseconds) OBT versus LAT blocks.** Mean response times of OBT versus LAT blocks plotted by Group: solid lines received active stimulation on Session 1 (*N* = 17; FBI-PT 1 and PT-FBI 1); dashed lines received active stimulation on Session 2 (*N* = 17; FBI-PT 2 and PT-FBI 2). *Stim* stimulation, *S1* session 1 (blocks 1–3), *S2* Session 2 (blocks 4–6), *OBT* own-body transformations, *LAT* left–right decision-making; *p*-value < 0.001 = *** over blocks (<***> between sessions).

First, we confirmed that participants responded faster on the control Lateralization task (*M* = 348 ms *SD* = 50 LAT) than on the more cognitively demanding Own-body Transformation task (*M* = 699 ms *SD* = 124 OBT). A strong learning effect on the OBT-task was found over consecutive blocks; this was not found on the control task, where participants had to make simple left–right judgments, *ps* < 1. A repeated MANOVA performed over the Own-body transformations demonstrated a significant improvement in response times over blocks, *F*(2,29) = 12.6, *p* < 0.001, ηp^2^ = 0.46, and between sessions, *F*(1,30) = 77.6, *p* < 0.001, ηp^2^ = 0.72, see Fig. 4. Furthermore, stimulation had a significant effect on OBT-task performance. As expected, this effect was in the opposite direction to that of the full-body illusion, making it harder to perform the self-other transformations. The first mixed ANOVA demonstrated a significant interaction between OBT response times and stimulation over blocks, Pillai’s Trace = 0.86, *F*(15,84) = 2.2, *p* = 0.011, ηp^2^ = 0.29. This interaction was also significant in the univariate within-subjects results, *F*(15,30) = 1.8, *p* = 0.047, ηp^2^ = 0.15. Overall response times had been longer when the right angular gyrus received active versus sham stimulation (Table 1). No significant group differences were found, *ps* < 1.

**Table 1:** Mean response times (milliseconds) OBT and LAT blocks.

OBT-task	Condition		M	SD	N	LAT-task	Condition		M	SD	N
**Session 1**						**Session 1**					
Block 1	1	FBI-PT Stimulation	789	181	9	Block 1	1	FBI-PT Stimulation	367	102	9
	2	FBI-PT Sham	779	127	8		2	FBI-PT Sham	319	21	8
	3	PT-FBI Stimulation	928	273	8		3	PT-FBI Stimulation	352	58	8
	4	PT-FBI Sham	829	155	9		4	PT-FBI Sham	379	63	9
Block 2	1	FBI-PT Stimulation	736	149	9	Block 2	1	FBI-PT Stimulation	346	42	9
	2	FBI-PT Sham	747	131	8		2	FBI-PT Sham	338	35	8
	3	PT-FBI Stimulation	777	175	8		3	PT-FBI Stimulation	329	37	8
	4	PT-FBI Sham	755	129	9		4	PT-FBI Sham	375	63	9
Block 3	1	FBI-PT Stimulation	706	142	9	Block 3	1	FBI-PT Stimulation	355	47	9
	2	FBI-PT Sham	677	126	8		2	FBI-PT Sham	341	65	8
	3	PT-FBI Stimulation	763	166	8		3	PT-FBI Stimulation	347	56	8
	4	PT-FBI Sham	711	106	9		4	PT-FBI Sham	393	74	9
**Session 2**						**Session 2**					
Block 1	1	FBI-PT Sham	696	96	9	Block 1	1	FBI-PT Sham	344	55	9
	2	FBI-PT Stimulation	621	55	8		2	FBI-PT Stimulation	319	21	8
	3	PT-FBI Sham	652	197	8		3	PT-FBI Sham	335	33	8
	4	PT-FBI Stimulation	649	113	9		4	PT-FBI Stimulation	377	68	9
Block 2	1	FBI-PT Sham	646	75	9	Block 2	1	FBI-PT Sham	339	46	9
	2	FBI-PT Stimulation	626	96	8		2	FBI-PT Stimulation	330	24	8
	3	PT-FBI Sham	613	93	8		3	PT-FBI Sham	333	30	8
	4	PT-FBI Stimulation	616	83	9		4	PT-FBI Stimulation	348	35	9
Block 3	1	FBI-PT Sham	648	82	9	Block 3	1	FBI-PT Sham	339	44	9
	2	FBI-PT Stimulation	567	48	8		2	FBI-PT Stimulation	327	21	8
	3	PT-FBI Sham	610	74	8		3	PT-FBI Sham	338	36	8
	4	PT- FBI Stimulation	641	99	9		4	PT-FBI Stimulation	359	36	9

Mean response time (M), standard deviation (SD), and population size (N) reported per experimental condition (1 FBI-PT; 2 FBI-PT; 3 PT-FBI; 4 PT-FBI) for OBT and control LAT blocks.

*FBI* full-body illusion, *PT* perspective taking task, *OBT* own-body transformations, *LAT* left–right decision-making.

Largely similar results were obtained when examining the accuracy of the Own-body Transformations (i.e., number of correct responses). A repeated MANOVA demonstrated a significant improvement in accuracy over sessions, *F*(1,30) = 7.8, *p* = 0.009, ηp^2^ = 0.21; while no improvement was found on the control task, *ps* < 1. Importantly, a mixed ANOVA performed over blocks found a significant interaction between the accuracy in Own-body Transformations and stimulation, *F*(15,74) = 2.2, *p* = 0.015, ηp^2^ = 0.31. This interaction was marginally significant in the univariate within-subjects results, *F*(6,60) = 2.0 , *p* = 0.079, ηp^2^ = 0.17. No interactions were found between the accuracy on the control task and stimulation, *ps* < 1.

## Discussion

Self-consciousness, in its most elementary form, may be disconnected from the phenomenal body^1,2^. Patients with psychosis frequently misattribute their thoughts and actions to external sources; and in certain out-of-body experiences, lucid states, and dreams body-ownership is absent but self-identification is preserved. A common factor between these experiences appears to be abnormal activation of the right angular gyrus, but its role in self-identification remained unclear. We hypothesized that self-identification depends on inferring self-location at the right angular gyrus (perspective-taking). This process relates to the discrimination of self-produced signals (endogenous attention) from environmental stimulation (exogenous attention^13^; Fig. 1). In a fully randomized, double-blind and sham-controlled HD-tDCS experiment we found a clear causal relation between right angular gyrus activation and alterations in self-location. Confirming our hypothesis, anodal versus sham HD-tDCS (i.e., stimulating the ‘other’ perspective) resulted in: (1) a more profound out-of-body shift in a full-body illusion; and (2) a reduction in people’s ability to discriminate self from other perspectives. Disembodiment appeared to be specifically characterized by an initial loss in sense-of-agency (i.e., decoupling of endogenous attention), but not in self-identification. Healthy volunteers controlled for susceptibility factors consistently reported: (1) more displacement towards the virtual body in centimeters; and (2) stronger out-of-body experiences when receiving active versus sham HD-tDCS. Furthermore, the stronger the self-identification with the virtual ‘other,’ the more displacement in centimeters was reported (i.e., alteration in self-location). Correspondingly, anodal versus sham HD-tDCS led to longer response times and less accuracy in judging self from other perspectives. In short, our results confirm a causal role of the right angular gyrus in spatiotemporal self-location (perspective-taking). Self-identification can be (functionally) decoupled from the bodily self, explaining phenomena associated with disembodiment.

The brain’s ability to locate itself (e.g.^8^) might be the key mechanism for self-identification, and distinguishing self from other signals (i.e., perspective taking). Recent evidence suggests that the brains’ ability to accurately locate itself is closely tied to the discrimination of self and other signals. Distinct attention networks appear responsible for the processing of endogenous (self) versus exogenous (other) signals^13^, and this allows the brain to keep track of different perspectives. As was addressed in the Introduction, in order to adequately process self-produced signals (i.e., endogenous attention) the brain needs to actively inhibit external stimulation (i.e., exogenous attention). This mechanism is inextricably tied to the predictability of self-produced signals, i.e., sense of agency. Previous studies have shown that when self-produced signals are recognized as one’s own, the right angular gyrus is inhibited by the frontal control network^15–18^. However, when this decoupling fails, voluntary movements are experienced as not controlled by oneself (see *movement disorders*^28^) and sometimes even misattributed as being caused by external sources (e.g., *delusion of control*^4^). In other words, not only can abnormal sensory processing lead to altered sensations and perceptions, but it can even provoke aberrant self-other reference frames, and, in more extreme cases, unusual self-location (Fig. 1). Out-of-body experiences and full-body illusions are unusual instances where self-produced signals are temporarily interrupted, while the right angular gyrus is (over)stimulated (^3,6,7^ and this study). Together, these findings suggest that disembodied reference frames result from a failure of the brain to recognize one’s own body’s signals, an *agency dysfunction*. Interestingly, still, the self as the center of awareness remains preserved. In this context, the proposed bidirectional neural mechanism offers a clear picture of how it is that self-identification can persist when the phenomenal body appears absent^1,2^.

Altered perceptions like those seen in psychosis and out-of-body experiences reveal a fundamental role of the right angular gyrus. There seem to be different ways to identify with the self: one directly related to the bodily self and the predictability of self-produced signals (i.e., frontal control network); and another, more reflective way of relating to the world and someone else’s perspective (i.e., right angular gyrus). How the brain directs its attention appears to be a fundamental aspect of the phenomenal self that has previously been overlooked. The respective roles of the dorsal and ventral attention systems in these processes need further examination (Fig. 1). The present study sought to explain the unusual phenomena associated with disembodiment by elucidating the role of the right angular gyrus in self-identification (i.e., perspective-taking). In this light, the present results could offer important future directions for developing more successful therapies to relieve psychosis symptoms and new rehabilitation techniques to aid in the recovery from a nervous system injury (e.g., spinal cord injury, brain damage, limb loss). One of the most challenging issues in neurorehabilitation is to establish accurate relationships between sensory perception and stored body representations^34^. In that context, future rehabilitation programs could benefit from the inclusion of experimental tasks and/or neurostimulation aimed at regaining proper right angular gyrus function and related network activity. This approach might result in a better balance between sensory processing and body representation, restoring proper sensorimotor loops and self-identification. At present, these and other clinical findings emphasize the critical involvement of the right angular gyrus when self-produced signals are no longer recognized (i.e., impaired endogenous attention). Together, these findings demonstrate why a breakdown in angular gyrus’ function seems to be particularly detrimental for (bodily) self-consciousness, e.g.: then we can no longer recognize ourselves in the mirror^10^; we feel ourselves disconnected from our own bodies and body parts (e.g.^8^); and the sharp distinction between our bodily selves and others is lost from us (e.g.^4^). However, the most fundamental aspect of ourselves appears to stay with us: the sense of being a discrete entity localized in the here (space) and now (time), perceiving the world from an immersive, first-person perspective^1,2,8^. Combining a full-body illusion paradigm with brain stimulation provided the first causal evidence in that direction.

## Conclusion

The present study reports a clear causal relation between right angular gyrus activation and alterations in spatiotemporal self-location (perspective-taking). Self-identification can be decoupled from the bodily self, explaining phenomena associated with disembodiment.

## Materials and methods

### Experimental design

This study comprised a fully randomized, double-blind and sham-controlled HD-tDCS experiment. The experiment had a crossover, mixed factorial design with two repeated measures (i.e., sessions) on the dependent variables and one independent grouping factor with four levels (the four experimental conditions, see “Results”). In each session, healthy volunteers controlled for susceptibility factors (see “Participants”) completed a (1) Full-body Illusion (FBI) paradigm and (2) a (control) Perspective Taking (PT) task. Half of the participants received active stimulation on Session 1versus sham stimulation on Session 2 (see “HD-tDCS” section and double-blinding procedure). The FBI-paradigm had two dependent variables: (1) the pre- and posttest displacement scores (*behavioral measure* with two levels) and (2) the total exit-interview scores (*psychometric measure*). The PT-task (composed of an OBT-task and control LAT-task) had four dependent variables with three levels: (1) mean response times and (2) accuracy scores were calculated for each of three blocks per task (see “Procedure and tasks”).

The expectations were as follows: all pretest displacement scores will measure 0 cm (i.e., no displacement); irrespective of task order or stimulation order, anodal (vs. sham) right angular gyrus stimulation will cause (1) more displacement towards the virtual body in centimeters (*behavioral measure*); and (2) stronger reported experiences on the exit-interview (*psychometric measure*); whereas (3) causing a reduction in discriminating self from other perspectives in reaction times and accuracy scores (note: this should not happen on the control LAT-task; overall RTs will be shorter). Furthermore, (4) behavioral and psychometric measures will be positively correlated (i.e., more displacement in centimeters means a stronger illusion); and (5) the reported displacement will be characterized by a loss in sense of agency, but not in self-identification (exit-interview; inter-item correlations *α* should be moderate to strong). Finally, some learning effects are anticipated between session 1 and 2 on the FBI-paradigm and session 1 (blocks 1–3) and 2 (blocks 4–6) on the OBT-task (note: this should not occur on the control LAT-task).

### Participants

Thirty-six naive right-handed adults with normal vision, hearing and vestibular function volunteered for the experiment (24 females, 12 males; mean age = 24.7; SD = 6.1). All received reimbursement in the form of course credit or gift cards. Since this was the first time a FBI-paradigm was combined with brain stimulation it was not possible to perform an a priori test to estimate group sizes. Therefore, this was estimated based on *N* sizes reported in similar neurostimulation studies, and taking into account the need to counterbalance four experimental groups (see^9^). Participants were recruited through an online research participant pool of the Queensland University of Technology (QUT) and study advertisements sent to staff email addresses (Brisbane, Australia). Potential participants were prescreened online for (1) age, (2) handedness^35^, (3) tDCS safety^36,37^, (4) pre-existing susceptibility to body illusions^38^, (5) (history of) psychological or neurological disorders, and (6) visual or vestibular problems viewing 3D content. Two participants reported prior Out-of-Body Experience during childhood (unrelated to a medical injury or near death experience). Susceptibility was assessed with the 20-item *time Perceptual Aberration Scale* (tPAS) and identified no individuals scoring within the top 10% of the scale. Past research found a strong correlation between the tPAS and the Perceptual Aberration Scale (i.e., measuring vulnerability to schizophrenia spectrum disorders), and between high tPAS scores and right angular gyrus (AG) dysfunction^38^. See Table S1 for a full list of exclusion criteria. Participants gave written informed consent and performed the experiment twice in two sessions scheduled one week apart. Informed consent to publish identifying images was obtained. This study was approved by the University Human Research Ethics Committee (QUT-UHREC) and conducted according to the Declaration of Helsinki (WMA, version October 2013).

### HD-tDCS

Stimulation was administered to the right angular gyrus (rAG) using a one-channel direct current stimulator (DC Stimulator Plus, NeuroConn) and a concentric centre-ring montage with two rubber electrodes^39,40^. A small circular electrode (diameter: 3 cm) served as the anode and was placed over the target region (rAG); while a concentric ring return electrode (inner/outer diameter: 7.5/10 cm) was placed evenly spaced around the anode. Electrodes were held into position with electroconductive paste (Ten20, Weaver) and an EEG-cap to ensure consistent adhesion to the scalp. The position of the centre electrode was determined using the 10–10 International EEG system (right AG, P4-P6; Brodmann Area 39), Fig. S1. Unlike conventional tDCS, where the current is projected between two large and distant electrodes, the concentric centre-ring montage constraints current flow safely to the target regions as demonstrated in previous modeling studies^12,39,40^. Recent studies have reliably measured regional^12,39^ and task-specific^12^ behavioral modulation with this set-up. In two counterbalanced sessions participants received either 25 min (1500 s) of anodal stimulation or “sham” stimulation to the rAG. In both sessions the current was gradually ramped up and down to 1 mA (50 s). This elicited a physical sensation on the scalp to ensure blinding of the participants, but did not modulate neural function in the sham stimulation condition. Blinding of the researchers was achieved by using the ‘study mode’ of the DC-Stimulator (a preassigned code triggered one of two stimulation conditions). To assure safe administration and blinding, participants rated the intensity of 11 potential adverse effects in each session (e.g., ‘*Headache*’ ranging from ‘*1* = *Absent*,’ ‘*2* = *Mild*,’ ‘*3* = *Moderate*,’ to ‘*4* = *Severe*’), for details see Table S1^41^. Both the participants and experimenter were unable to guess stimulation order, respectively: (1) when the manipulation was revealed at study completion; and (2) based on differences, e.g., in skin redness (which were absent). Sessions were held a week apart to avoid carryover effects.

### 3D live-streaming full-body illusion paradigm

A new 3D full-body illusion (FBI) was developed that included a montage of two action cameras to capture and live-stream 3D-images that were in real-time projected onto a large screen. The video cameras live-captured participants from behind, while they looked at themselves being stroked on the back projected live-size in 3D in front of them. Therefore, participants could ‘observe’ in front of them what they ‘felt’ happening to them. This created the illusion of an Out-of-Body Experience; see Fig. S2 for the experimental setup. Furthermore, with the use of plastic 3D-goggles, for the first time participants were able to observe the illusion while simultaneous stimulation could be administered safely. (Note: it was not possible to recreate an FBI using a VR-paradigm and Head-mounted Display. The electrical stimulation of the brain would interfere with the electronic equipment and this could potentially be unsafe.) The equipment used for the live stereoscopic 3D-streaming consisted of a set of two 3D-mounted action cameras (brand: Xiaomi Yi; 2 K resolution) that were connected to an ASUS ROG Strix gaming laptop by two AGPtek USB 3.0 capture cards. In return, the gaming laptop was connected via a high-speed HDMI cable to an Optoma HD 3D-projector. At 60 frames per second it projected full HD stereoscopic 3D images onto a white screen positioned four meters away and two meters in front of the observer. To ensure the best quality projection, the room was kept dark except for two spotlights that illuminated participants’ backs from opposite directions (surroundings were blacked out). Real-time merging of the two separate video streams, and any necessary adjustments (e.g., image-size corrections; left camera horizontal flip; zooming of streams), were done in Bino 3D player-software 1.6.7^42^ using a Linux operating system (Ubuntu 18.04 LTS software with kernel 4.19.5).

### Procedure and tasks

At the start of the experiment participants either sat comfortably behind a desk at arm’s length distance from a 17.3 in. 1920 × 1080 resolution laptop screen (PT); or two meters from a large projection screen (FBI; the laptop was removed during this task). They were instructed to sit motionless for the duration of the tasks. Short breaks were inserted between the tasks and blocks to take time to rest.

For (A) *the Self-Other Perspective Taking task* the classical Own-body Transformation (OBT) versus control Lateralization (LAT) task was chosen^33^. In the OBT-task participants saw sequences of either front-faced or back-faced human figures and were required to judge whether one of the marked hands (i.e., appearing to wear a grey glove) was the right or left hand of the figure, see Fig. S3 for task stimuli A–D. To ensure that the participants did not adopt a strategy, they were instructed to perform the mental transformations on each trial by imagining themselves in the position of the figure and only then to make a response. Participants completed a short 60 s trial session before commencing the actual task (eight trials). The OBT task consisted of three blocks of 80 items each. In each block, one of the four stimuli (A–D) appeared 20 times in a randomized order. Stimuli were shown in the center of the screen (5.0° × 6.1° visual angle) for a duration of 200 ms. In between a centered fixation cross could be observed (1000 ms ISI). Participants were instructed to keep focusing on the fixation cross and upon seeing the figure to make a response as quickly and accurately as possible. Responses were made with the right hand using the index (‘<’ left arrow key) and middle fingers (‘>’ right arrow key). In a control condition, i.e., *the lateralization task*, the same visual stimuli were presented in three blocks. However, this time participants only had to indicate on which side of the screen the marked hand was shown (without performing mental transformations). The OBT and LAT tasks are known to only differ from each other in the ‘own-body transformations,’ i.e., in mental rotation and Self-Other perspective^43^. Therefore, tasks were identical except for the degree of transformations required. The perspective switching task lasted 10–15 min and ran on PsychoPy v1.90.3^44^.

To standardize (B) *the Full-body Illusion* between experimental sessions participants wore a white T-shirt with no prints on the back. Upon arrival, they were invited to comfortably sit down on a stool behind a large table located in the middle of the room. The table was positioned about 200 cm from a large white projection screen. In the middle of the table, directly in front of the stool, a measuring tape was visible that stretched out over the total width of the table. Hence, the tape started counting 0 cm from the participants’ body out towards the screen. First, participants were asked to indicate their perceived location on the measuring tape (i.e., pretest score). Subsequently, they were asked to wear 3D goggles and place their hands loosely on top of the table. Their hands were covered by a black sheet that matched a black table cloth that covered the table. Before beginning, participants were instructed to keep completely still during the illusion and to focus on what was happening in front of them. In the following 8 min participants were stroked on the back (with a brush fastened on top of a stick) while this was live-captured from behind and the images in real-time projected in front of them. The stroking centered on the upper part of the middle back over a length of 20 cm and was carefully executed at a pace of 50 strokes per minute (i.e., the researcher had received previous training with a metronome). In short, during a continuous period of eight minutes participants could ‘observe’ in front of them what they ‘felt’ happening to them.

The effectiveness of the FBI was measured in two-fold: (1) ‘*Displacement scores*’ were taken before (pretest score) and after the illusion (posttest score) using the measuring tape fixed onto the desk in front of the participants. In case participants indicated to have felt a displacement towards the virtual body, they were asked to point out in centimeters to what extent they had felt the displacement. Pointing ‘0 cm’ on the tape localized participants to their body (i.e., no displacement); while pointing ‘200 cm’ on the tape localized participants to the virtual body (range 0–200 cm). Directly after the illusion (2) *an exit-interview* was completed that explicitly asked participants about their experience (5 min duration). Participants indicated their agreement to 15 statements intended to measure displacement, e.g., ‘*I felt I was in front of my body*,’ scored on 5-point Likert scales ranging from ‘*1* = *strongly disagree*’ to ‘*5* = *strongly agree*,’ see Table S3 for a full list of statements. In addition, they were asked to estimate (a) the onset time of the displacement from ‘*0* = *never*,’ ‘*1* = *after a while*’ to ‘*2* = *fairly quickly*,’ (b) the onset time in minutes, and (c) the frequency of the displacement from ‘*0* = *never*,’ ‘*1* = *once shortly*,’ *2* = *many short times*’ to ‘*3* = *continuously.*’ In completion, participants provided a written description of their experience. The next week participants returned and completed the same routine. Each session lasted ~ 90 min and was scheduled approximately at the same time of day.

### Statistical analysis

The experiment had six dependent variables spread over two tasks (Full-body Illusion FBI; Perspective Taking PT) with two repeated measures (Session 1 and 2) and one grouping variable (the four experimental conditions, see “Results”). Each of the dependent variables (e.g., “*Total Exit Interview Score*”) was analyzed separately in a mixed ANOVA with one between-subjects factor (e.g., “*Experimental Condition*” with four levels) and one within-subjects factor (e.g., “*Session*” with two levels). Where appropriate, the four experimental groups were taken together creating an independent variable “*Stimulation Order*” with two levels: stimulation Session 1 (FBI-PT 1 and PT-FBI 1) versus stimulation Session 2 (FBI-PT 2 and PT-FBI 2). In addition, the dependent variables of the PT-task were analyzed in a one-way repeated measures MANOVA including an additional dependent variable for “*Time*” to check for task-learning effects over consecutive blocks and sessions irrespective of stimulation order.

## Supplementary information

Supplementary Information.

## Data Availability

Data from consenting participants have been deposited to 10.17605/OSF.IO/B9TKU. All data is available in the main text or the supplementary materials.
