# Author Correction: A causal role for the right angular gyrus in self-location mediated perspective taking

**DOI:** 10.1038/s41598-021-83014-5

**Published:** 2021-02-09

**Authors:** D. M. L. de Boer, P. J. Johnston, G. Kerr, M. Meinzer, A. Cleeremans

**Affiliations:** 1 School of Psychology and Counselling, Faculty of Health, Queensland University of Technology (QUT), Kelvin Grove, QLD 4059 Australia; 2 School of Exercise and Nutrition Sciences, Faculty of Health, Queensland University of Technology (QUT), Kelvin Grove, QLD 4059 Australia; 3 Institute of Health and Biomedical Innovation (IHBI), Queensland University of Technology (QUT), Brisbane, Australia; 4 Centre for Clinical Research (UQCCR), The University of Queensland, Brisbane, Australia; 5 Department of Neurology, University Medicine Greifswald, Greifswald, Germany; 6 Consciousness, Cognition, and Computation Group (CO3), Centre for Research in Cognition and Neurosciences (CRCN), ULB Neuroscience Institute (UNI), Université Libre de Bruxelles (ULB), Avenue F.D. Roosevelt 50, CP191, 1050 Brussels, Belgium

Correction to: *Scientific Reports* https://www.doi.org/10.1038/s41598-020-76235-7, published online 05 November 2020

The Supplementary Information published with this Article contains errors. The reference numbers do not correspond to the correct references in the original version of the Article.

In addition, the Supplementary Information contains a rounding error, where

“Inter-item correlations measured with Cronbach’s α: 0.89 Session 1; 0.87 Session 2 (displacement items); 0.81 Session 1; 0.79 Session 2 (15 items excl. control items 1, 9 & 14).”

should read:

“Inter-item correlations measured with Cronbach’s α: 0.89 Session 1; 0.87 Session 2 (displacement items); 0.81 Session 1; 0.80 Session 2 (15 items excl. control items 1, 9 & 14).”

The correct Supplementary Information file is provided below.

## Supplementary Information

Supplementary Information.

