# Supplementary material for: Author Correction: A causal role for the right angular gyrus in self-location mediated perspective taking
Source: Sci Rep. 2021 Feb 9;11:3819. doi: 10.1038/s41598-021-83014-5 (PMC7873186; doi:10.1038/s41598-021-83014-5)
Supplement: Supplementary file 1 — Supplementary Information. [file 41598_2021_83014_MOESM1_ESM.docx]

Supplementary Materials for

*A causal role for the right Angular Gyrus in Self-Location mediated Perspective Taking*

D.M.L. de Boer^1,3^*, P.J. Johnston^1,3^, G. Kerr^2,3^, M. Meinzer^4,5^, A. Cleeremans^6^.

Correspondence to: [debbie.boer@hdr.qut.edu.au](mailto:debbie.boer@hdr.qut.edu.au)

**This file includes:**

Tables S1 to S3 *(note: 300dpi images in separate files)*

Figs. S1 to S3 *(note: 300dpi images in separate files)*

Data S1

**Other Supplementary Materials for this manuscript include the following:**

Data S2 (OSF)


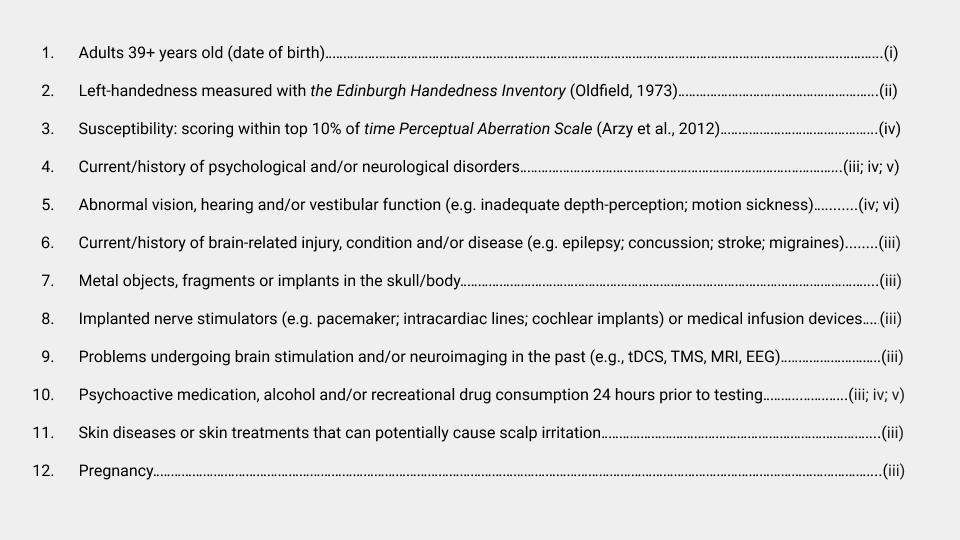


**Table S1. Exclusion Criteria.**

Prospective participants were prescreened online for (i) age, (ii) handedness, (iii) tDCS Safety, (iv) pre-existing susceptibility to body illusions / right Angular Gyrus dysfunction *38*, (v) (history of) psychological and/or neurological disorders, and (vi) visual or vestibular problems viewing 3D content (corrected-to-normal vision was allowed using contact lenses). Participants’ eligibility was double-checked prior to testing (e.g., date of birth, check 3D vision, *tDCS Safety Screening Questionnaire* *36-37*; *Edinburgh Handedness Inventory* *35*). Further restrictions aimed to control for declines in reaction times with age and left-right differences in hemispheric organization.


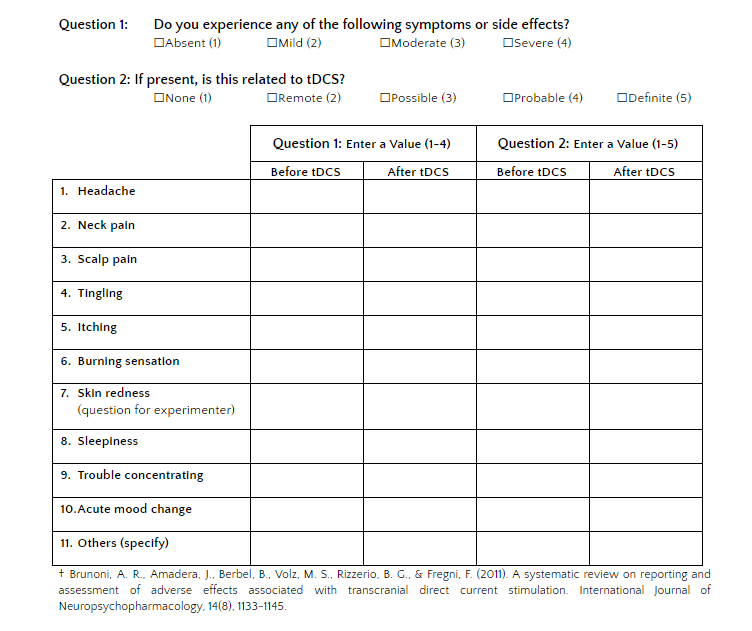


**Table S2. Adverse Effects tDCS.**

Participants rated the intensity of 11 potential adverse effects before and after each tDCS session, e.g., *‘Headache’* ranging from *‘1= Absent,’ ‘2 = Mild,’ ‘3 = Moderate’* to *‘4 = Severe’* (*41*). Questions 1 & 2 were compared to identify potential symptoms that were related to the tDCS administration. This protocol assured safe tDCS administration and blinding to stimulation condition.


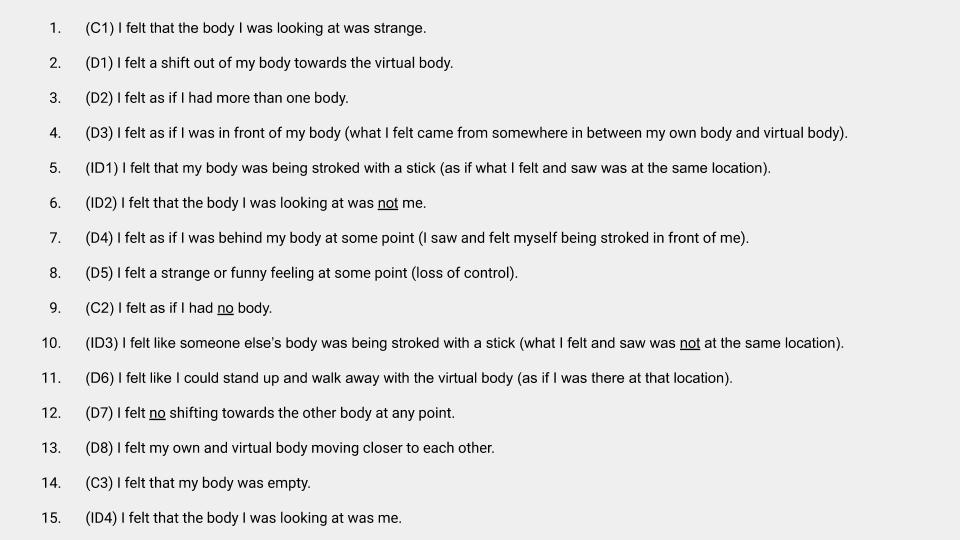


**Table S3. Full-body Illusion (FBI) Exit Interview.**

Listed items were answered and scored on 5-point Likert scales ranging from ‘*5 = Strongly Agree*,’ ‘*4 = Agree*,’ ‘*3 = Neither agree or disagree*,’ ‘*2 = Disagree*’ to ‘*1 = Strongly Disagree’* (negatively phrased items reverse-coded); Item Codes: D1-8 Displacement; ID1-4 Self-Identification; C1-3 Control (C1 measured ‘General Disposition’ to illusion between sessions); D5 & D6 measured changes in perceived control, i.e., ‘Sense of Agency’ (answers were verified with response to open question: ‘*Please describe your experience in a few words*’).


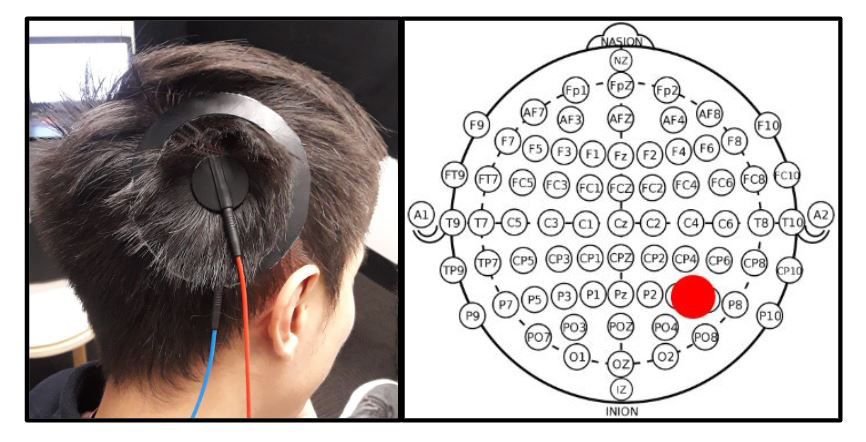


**Fig. S1. HD-tDCS montage & P4-P6 electrode positioning.**

       (L)   HD-tDCS concentric center-ring montage to right Angular Gyrus;

       (R)   P4-P6 electrode positioning (Brodmann Area 39) in 10-10 International EEG system.


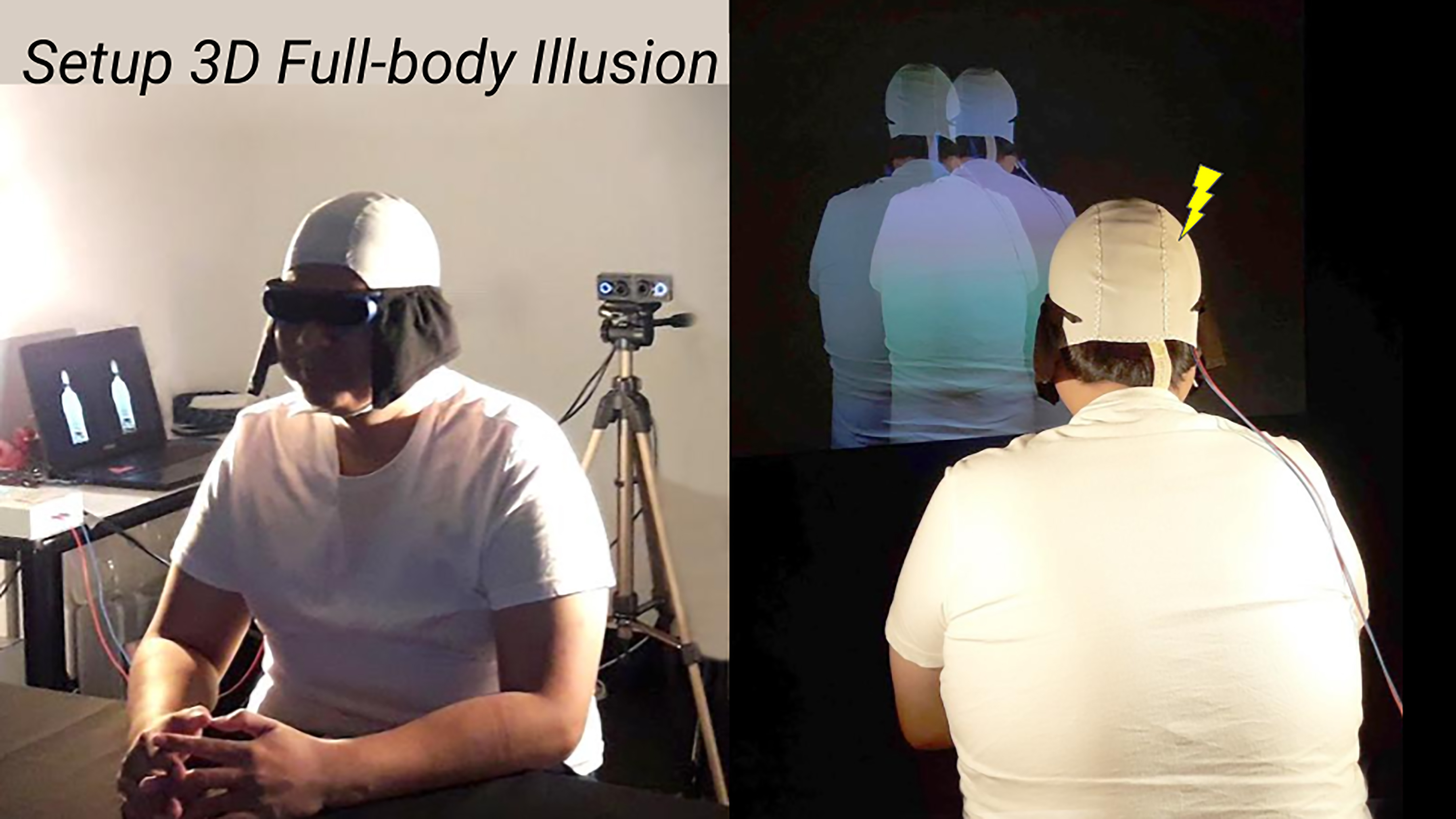


**Fig. S2. Stereoscopic 3D Full-body Illusion combined with HD-tDCS.**

Front (L) and back (R) view of Full-body Illusion paradigm based on live-streaming stereoscopic 3D-images with Bino 3D Player (*42*). Here combined with High Definition transcranial Direct Current Stimulation (HD-tDCS).


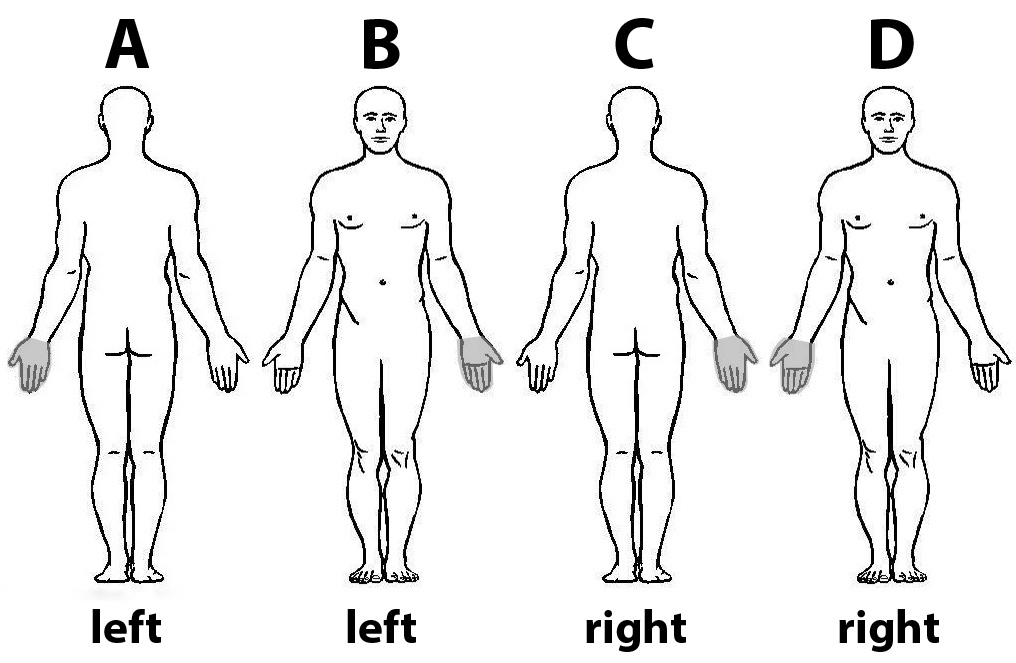


**Fig. S3. Own-body Transformation task (OBT & LAT) stimuli A-D.**

         Own-body Transformation and control Lateralization stimuli A-D; below correct answers OBT-task.

          Adapted from (*33*) including more realistic human sketches.

Data S1. Full-body Illusion (FBI) Exit Interview

The FBI exit-interview consisted of 15 items (incl. 3 control items) that were answered and scored on 5-point Likert scales. There were eight *‘Displacement Items,’* two of them measured sense-of-agency (i.e., *‘Agency low’* & *‘Agency high’*), and four *‘Self-Identification Items,’* see Table S3. Negatively phrased questions were reverse coded and did not indicate inconsistencies in answers. Subsequently, a high score on each item (excl. control items) represented a more pronounced full-body illusion. Descriptive statistics pooled over the sessions confirmed that all items (except control items 9 & 14) had a mean score around 3 points and SD > 1 point (except Item 15). As expected, the displacement items (Items 2, 3, 4, 7, 8, 11, 12 & 13) were strongly correlated, *r*(33)  > 0.5, *p* = 0.01; while other items were not correlated, *r*  = 0 one-tailed Bonferroni corrected. Inter-item correlations measured with Cronbach’s α: 0.89 Session 1; 0.87 Session 2 (displacement items); 0.81 Session 1; 0.80 Session 2 (15 items excl. control items 1, 9 & 14). *“Total Exit Interview Scores”* were calculated excluding the control items.

Data S2. (OSF) <https://doi.org/10.17605/OSF.IO/B9TKU>
